# Midwifery learning and forecasting: Predicting content demand with user-generated logs^☆^

**DOI:** 10.1016/j.artmed.2023.102511

**Published:** 2023-04

**Authors:** Anna Guitart, Ana Fernández del Río, África Periáñez, Lauren Bellhouse

**Affiliations:** a benshi.ai, Passeig de Gracia, 74, Barcelona 08008, Spain; b Maternity Foundation, Forbindelsesvej 3, 2. floor, Copenhagen 2100, Denmark

**Keywords:** Time series forecasting, Deep learning, mHealth, Maternal and neonatal care

## Abstract

Every day, 800 women and 6700 newborns die from complications related to pregnancy or childbirth. A well-trained midwife can prevent most of these maternal and newborn deaths. Data science models together with logs generated by users of online learning applications for midwives can help improve their learning competencies. In this work, we evaluate various forecasting methods to determine the future interest of users for the different types of content available in the Safe Delivery App, a digital training tool for skilled birth attendants, broken down by profession and region. This first attempt at health content demand forecasting for midwifery learning shows that DeepAR can accurately anticipate content demand in operational settings, and could therefore be used to offer users personalized content and to provide an adaptive learning journey.

## Highlights

• Online training tools can help midwives acquire critical skills.
• Time series forecasting methods can be used to predict content demand by user type.
• Content demand predictions can be used to design adaptive learning journeys.
• DeepAR is the best method of those evaluated to generate predictions in production.

## 1 Introduction

The rapid expansion of mobile health applications in low- and middle- income countries and the large volume of data generated by their users has created unprecedented opportunities for applying artificial intelligence (AI) to improve individual and population health [1], [2]. The application of data science models to the digital tools' behavioral logs of frontline healthcare workers and patients can lead to improvements in clinical research and practice and health service delivery. Moreover, public health can use big datasets to promote healthy habits and ameliorate self-management by providing people with health and well-being plans based on their medical and social circumstances [3], [4].

Every year 300,000 women and 5 million newborns die of causes related to pregnancy and childbirth [5], [6]. Additionally, for every maternal death, approximately 20 women suffer serious birth injuries [7]. Nearly all of these deaths and disabilities occur in low- and middle-income countries, and almost 90 % of them could be prevented if the woman gave birth with qualified assistance from a skilled midwife [8]. Additionally, 80 % of all newborn deaths result from conditions that are preventable and treatable and for which proven, cost-efficient interventions are available [9]. Almost all intrapartum and many antepartum stillbirths could be prevented with quality essential childbirth care and antenatal care [10].

Mobile health (mHealth) apps are valuable tools to communicate with users and collect large quantities of fine-grained high-frequency behavioral data describing the user activity. Consequently, mHealth apps can support data-driven precision nudging, allowing personalized interventions to be provided to the users depending on their situational needs. Therefore, in this study, we analyse the logs from skilled birth attendants using the *Safe Delivery App* [11], a digital training tool developed by Maternity Foundation, the University of Copenhagen, and the University of Southern Denmark that contains evidence-based obstetric and newborn guidelines for skilled birth attendants to ensure maternal and neonatal safety in childbirth [12]. This work represents a step towards content personalization for midwives, in a sector that has traditionally been left out of big technological developments. Forecasting the demand for learning content by profession and region can lead to a better understanding of user habits and improve the management of campaigns [13]. We apply several forecasting methods to evaluate their accuracy and production feasibility with the aim of using the outcomes for future experimentation and incentive analysis. Previous studies using similar methodologies and user logs can be found in [14], [15].

The relevance of capacity-building apps for healthcare workers' skill acquisition, and hence for medical practice and quality of care, has been specifically examined in the case of the Safe Delivery App for its impact on the maternal and neonatal care delivered. In [16], the results of a cluster randomized control trial involving 176 skilled birth attendants in 73 healthcare facilities in Ethiopia are analyzed. Those in treatment group received a smartphone with the Safe Delivery App. In the year and a half the study lasted, 3601 women went into active labor and were assisted by health workers participating in the control trial. Perinatal death occurrence among births attended by them and knowledge of neonatal resuscitation skills were assessed 6 and 12 months after the intervention, finding a non-significant reduction of perinatal mortality (14 vs. 23 per 1000 births) and a significant increase in skill scores at both 6 and 12 months (80 % and 107 % higher respectively) in participants that used the Safe Delivery App. An additional case study with this app in Ethiopia is described in [17], which also shows a consistent increase in skill and knowledge scores of the app's users.

### 1.1 Related work

Certain aspects of digital health solutions for improved health outcomes have been previously studied [18], [19], [20], [21], [22]. These studies demonstrated how digital tools can promote evidence-based design and evaluation of policies through health-driven interventions based on large quantities of behavioral and health-related data.

Focusing on low- and middle-income countries, several studies [20], [23], [24], [25] highlighted how digital tools can overcome resource scarcity and provide support for healthcare providers and patients, given the increasing mobile penetration in these areas.

In the context of healthcare e-learning, mobile training is essential [26] to overcome the shortage of health professionals worldwide. Mobile e-learning is expected to become increasingly more personalized and adaptive and emerge as a valuable means to deliver educational content based on the learners' needs [27]. Furthermore, mobile devices can help provide instant learning at the point of care. These considerations form the basis for our research objective, i.e., to develop a framework to facilitate the design of incentives to enable personalized training.

Several studies on mobile e-learning focused on user engagement and personalization through learner profiling and recommendations [28], [29], [30], [31]. Katsaris and Vidakis [30] reviewed the literature on adaptive e-learning systems and emphasized the importance and efficiency of using different learning profiles in the adaptive learning process. Other researchers combined gamification in mHealth learning [32], [33]. Entertainment games, user engagement, and content personalization and recommendation in mobile apps have also attracted considerable research attention [34], [35], [36].

A notable aspect of app and content personalization is predicting the demand of e-learning content, focusing on time series data. A straightforward example of personalization via content demand forecasting is to order the learning modules in the app according to decreasing predicted demand for each type of user. A more complex approach would be to use the output of the demand forecasting models as features for a machine learning recommender system. In this context, student learning behavioral data, which is typically time series data, have been studied and predicted. In general, each clickstream/in-app log of a student is accompanied by a timestamp and represents the learning or content demand in the digital framework. Consequently, online education prediction based on time series data has attracted extensive research attention [37]. Many researchers have focused on e-learning demand and user dropout prediction [38], [39], [40]. Notably, these studies used time series data only for establishing predictive models for classification or regression and not for developing forecasting models to predict future trends. Other studies have developed forecasting models based on neural network techniques to predict the future dropout and content demand in massive open online courses [41], [42], [43], [44], [45]. Additionally, the course performance and learning outcomes in online learning activities have been predicted using user behavioral data through time series forecasting [46], [47], [48], [49] and other machine learning models [37]. Namoun and Alshanqiti presented a comprehensive literature review on these aspects [50].

Forecasting is a key tool for predicting future health events or situational parameters such as demands for health services and healthcare needs [51]. Representative studies on time series demand forecasting for mHealth devices include [13], [52], [53], [54], [55], [56], which focused on use cases such as medical device demand and utilization or online medical consultation demand. Soyiri and Reidpath presented an overview of health forecasting research [51]. Outside the realm of mHealth, other recent studies using deep learning approaches to understand and predict health outcomes include [57], [58], [59], [60].

In addition to estimating the demand, the above mentioned models can shed light on user behavioral habits and facilitate the planning of future health training programs or events. In freemium mobile entertainment games, these models have been incorporated to realize in-game event simulations [14]. Furthermore, the outcomes of health forecasting can be used in intervention-based experimentation or simulations to reconfigure health services [61] and support decision-making in healthcare management [62].

### 1.2 Our contribution

To the best of our knowledge, this study represents the first attempt at combining demand forecasting with e-learning to predict health content demand for midwifery learning. We demonstrate the validity of this approach with user logs of the Safe Delivery App, and discuss how it could be used for planning, e-learning content personalization, and incentive-based intervention policies to promote its use. This work contributes to the research and operational application of machine learning models in the context of digital health. In particular, it deals with content personalization for capacity-building apps. Facilitating healthcare workers' skill acquisition will result in improved medical practice and enhanced quality of care.

## 2 Methods

We compare the performance of different time series forecasting methods in predicting the daily demand (in number of users) per type of e-learning content (*module*, in the app's language) and user's profession. Training and prediction were performed using the gluonTS [63], keras [64] and mxnet [65] Python libraries and the Forecast R package [66].

### 2.1 Seasonal naïve

The naïve forecast model [63] was used as a benchmark. Its forecasts are given by:

(1) $$\hat{y}\left(t+k\right)=y\left(t+k-h\right)$$

where *t* is the forecast time point, k refers to the prediction time points ranging all prediction lengths *k* = 0, 1, .., *prediction_length* − 1 and *h* is the length of the seasonal period. Predictions for each horizon are given by the exact values at the equivalent time points of the previous season. For prediction lengths larger than a season *h*, the season is repeated multiple times, whereas, for time series shorter than a season, the mean observed value is used as the prediction.

### 2.2 Seasonal autoregressive integrated moving average (SARIMA)

The SARIMA model [67] was used as an additional benchmark, as it is one of the best performing and most widely used classical approaches to time series analysis and forecasting. At each time step, the time series value is a combination of regular and seasonal autoregressive (where the value depends on the previous values) and moving average (where the value depends on the previous errors) polynomials. In addition, one can take as many differences as needed in the original time series to make it stationary.

The formula for the seasonal ARIMA is often represented as *ARIMA*(*p*, *d*, *q*)(*P*, *D*, *Q*)_*m*_, the first three terms representing the autoregressive *p*, differencing *d*, and moving average *q* polynomial order, respectively. The parameters, *D*, *Q* are the seasonal counterparts with the order of the seasonality being represented by *m* . The ARIMA model can be represented by the following equation:

(2) $$\varphi \left(L\right){\left(1-L\right)}^{d}y_{t}=\theta \left(L\right)\varepsilon_{t},$$

where *Lφ*_*t*_ = *φ*_*t*−1_, with *L* being the lag operator, sometimes also called backshift operator. Therefore, the seasonal counterpart SARIMA can then be represented as

(3) $$\varphi \left(L\right)\varphi \left(L^{m}\right){\left(1-L\right)}^{d}{\left(1-L^{m}\right)}^{D}y_{t}=\theta \left(L\right)\theta \left(L^{m}\right)\varepsilon_{t}$$

### 2.3 Neural networks with categorical embedding

The last decade has seen the rapid growth of deep neural network architectures to address a great variety of problems [68], driven by increasing computational resources, data availability, and improved methodologies. One shortcoming of this approach is the difficulty in including categorical features owing to the lack of continuity. Entity embedding [69] can be used to effectively learn the representation of categorical variables in multidimensional spaces, thereby increasing their continuity and providing an intelligent way of using them as features in deep learning models. In particular, such networks can overcome the problems faced by the more traditional approach of one-hot encoding, specifically, the need for excessive computational power and tendency towards overfitting.

Let's denote the mapping of the discrete categorical variable *x*_*i*_ to a vector by

(4) $$x_{i}\to x_{i}.$$

Let's also denote the mapping of the same variable *x*_*i*_ to a one-hot encoding as

(5) $$x_{i}\to \delta_{x_{i}\alpha },$$

where *δ*_*x*_*i*_*α*_ is the Kronecker delta, a vector of the length equal to the number of values of the categorical variable *x*_*i*_ with all components zero except for *α* = *x*_*i*_, where it takes value one.

Adding an extra layer of linear neurons on top of the one-hot encoded input in Eq. (5) is equivalent to the mapping of the categorical variable to a vector in Eq. (4). This results in the output of the extra layer of linear neurons, *x*_*i*_, that can be expressed as

(6) $$x_{i}\equiv \sum_{\alpha }w_{\alpha \beta }\delta_{x_{i}\alpha }\equiv w_{x_{i}\beta }$$

where *w*_*αβ*_ is the weight connecting the one-hot encoding layer to the embedding layer and *β* is the index of the embedding layer. If there is more than one categorical variable, all embedding layers and the input of all continuous variables are then concatenated. The final merged layer is treated like a normal input layer of neural networks, and additional layers can be built on top of it. For further details see [69].

### 2.4 Autoregressive recurrent networks (DeepAR)

The use of autoregressive recurrent networks to simultaneously predict many time series was introduced in [70]. It is a state-of-the-art well established probabilistic time series forecasting method that has been shown to outperform traditional baselines and many recently developed deep learning based forecasting methods (see [71] for a recent survey with examples).

The method trains either long short-term memory (LSTM) or gated recurrent unit (GRU) networks, where the inputs at each time step are the covariates, the target value from the previous time step (which makes it autoregressive), and the previous output of the network (which makes it recurrent). A global model is learned from all the time series that can be used to generate probabilistic forecasts for the individual time series, each with its own individual distribution. This technique has been previously used in connection with healthcare in [72].

The conditional distribution of each time series *i* and its values denoted as *z*_*i*, *t*_ for each time *t* , is represented by

(7) $$P\left(z_{i,t_{0}:T}|z_{i,1:t_{0-1}},x_{i,1:T}\right)$$

where *t*_0_ denotes the time point from which we assume *z*_*i*, *t*_ to be unknown at prediction time, and *x*_*i*, 1:*T*_ are covariates that are assumed to be known for all time points. The target/objective is to model the future (prediction range) of each time series values, represented as [*z*_*i*, *t*−0_, *z*_*i*, *t*_0_+1_, …, *z*_*i*, *T*_] from their past values (conditioning range) [*z*_*i*, 1_, *z*_*i*, *t*_0_+1_, …, *z*_*i*, *t*_0_−1_]. This approach assumes that the previous model distribution consists of a product of likelihood factors parametrized by the output *h*_*i*, *t*_ of an autoregressive recurrent network, as shown in 8. *h* is a function implemented by a multi-layer recurrent neural network with LSTM cell, se Eq. (9).

(8) $$P\left(z_{i,t_{0}:T}|z_{i,1:t_{0-1}},x_{i,1:T}\right)=Q_{\theta }\left(z_{i,t_{0}:T}|z_{i,1:t_{0-1}},x_{i,1:T}\right)=\prod_{t=t_{0}}^{T}l\left(z_{i,t}|\theta \left(h_{i,T},\Theta \right)\right)$$

(9) $$h_{i,t}=h\left(h_{i,t-1},z_{i,t-1},x_{i,t},\Theta \right)$$

where the likelihood *l*(*z*_*i*, *t*_| *θ*(*h*_*i*, *T*_)) is a fixed distribution whose parameters are given by a function *θ*(*h*_*i*, *t*_, Θ) of the network output *h*_*i*, *t*_. For more details see [70].

The network directly predicts all parameters Θ (e.g. mean and variance) of the probability distribution for the next time point.

The distributions are parametrized, given the output of the neural network system/architercture. For modeling time series of positive count data, the negative binomial distribution *l*_*NB*_ is a commonly used choice, as is the one we selected for this work. The parametrization of this distribution is then given by the mean *μ* and a shape parameter *α* both parameters obtained from the network output by a fully-connected layer with soft-plus activation to ensure positivity. See the parametrization of the negative binomial distribution below

(10) $$l_{\mathrm{NB}}\left(z|\mu ,\alpha \right)=\frac{\Gamma \left(z+\frac{1}{\alpha }\right)}{\Gamma \left(z+1\right)\Gamma \left(\frac{1}{\alpha }\right)}{\left(\frac{1}{1+\alpha \mu }\right)}^{\frac{1}{\alpha }}{\left(\frac{\alpha \mu }{1+\alpha \mu }\right)}^{z}$$

(11) $$\mu \left(h_{i,t}\right)=\log\left(1+\exp\left(w_{\mu }^{T}h_{i,t}+b_{\mu }\right)\right)$$

(12) $$\alpha \left(h_{i,t}\right)=\log\left(1+\exp\left(w_{\mu }^{T}h_{i,t}+b_{\alpha }\right)\right)$$

where *μ* ∈ *ℝ* and *α* ∈ *ℝ*.

### 2.5 Low-rank Gaussian copula processes

This is a multivariate approach with deep learning elements, described as GP-Copula in [73], and is another state-of-the-art probabilistic time series forecasting method [71]. It combines a time series model based on autoregressive recurrent networks with a Gaussian copula process to parameterize the output distribution. This copula has a low-rank structure to keep the number of parameters and computational complexity within reasonable bounds.

The joint distribution of the observations is factorized by

(13) $$p\left(z_{1},\ldots ,z_{T+\theta }\right)=\prod_{t=1}^{T+\theta }p\left(z_{t}|z_{1},..,z_{t-1}\right)=\prod_{t=1}^{T+\theta }p\left(z_{t}|h_{t}\right)$$

The parameters *θ* are learnt from the observed data [*z*_1_, …, *z*_*T*_] by minimizing the loss function

(14) $$-\log p\left(z_{1},\ldots ,z_{T}\right)=\sum_{t=1}^{T}\log p\left(z_{t}|h_{t}\right)$$

with stochastic gradient descent-based optimization.

## 3 Modeling

### 3.1 Dataset

Our dataset is composed of user logs extracted from Maternity Foundation's *Safe Delivery App*. This app targets skilled birth attendants around the world, empowering them to provide a safer birth for mothers and newborns through evidence-based and up-to-date clinical guidelines on maternal and neonatal care, including the core components or “signal functions” of basic emergency obstetric and newborn care.

*Safe Delivery App* involves several learning topics known as modules: *Active Management of Third Stage Labour*, *Female Genital Mutilation*, *Hypertension*, *Infection Prevention*, *Manual Removal of Placenta*, *Maternal Sepsis*, *Neonatal Resuscitation*, *Newborn Management*, *Post Abortion Care* and *Postpartum Hemorrhage*. The possible professions of the app users (as self-reported by them on first login) are *Midwife*, *Nurse*, *Other skilled birth attendant*, *Physician* or *Student*.

**Fig. 1:**
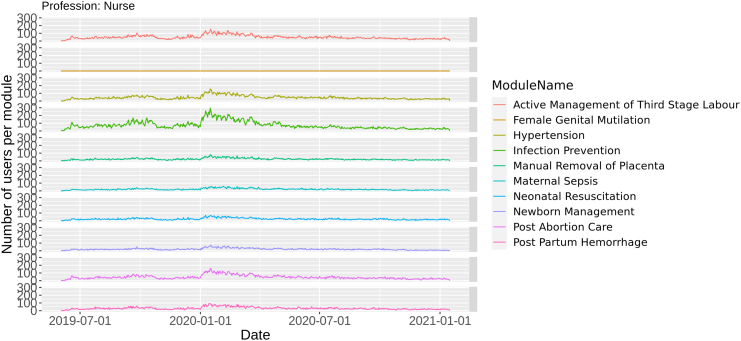
*Nurses* module usage. Number of daily users who accessed a particular *Safe Delivery App* module, for India *nurses*.

Data were collected from 2019-05-01 to 2021-01-14, and the results shown correspond to a sample of 20,422 users from India. We built daily time series of module usage per profession, showing the number of professionals that accessed a particular module per day and took them as a proxy for the demand for that specific content. Fig. 1 presents the time series for various modules in the case of *nurses*. Even though all modules show a similar overall usage trend, each series exhibits different scale and usage patterns. Similar series are obtained for the other professions. Our goal is to predict the app usage per profession to personalize the content and better grasp the usage dynamics.

### 3.2 Model specification

The goal was to predict the daily values of the usage time series for each month and each module-profession combination, training the model with all the available data until the end of the previous month. Cross-validation was performed using a rolling window [74], [75] from 2020-06-01 to 2020-12-01, considering all historical data before the prediction date for the training samples and 30 days of data before the prediction date for the test sample (training samples were split into training and validation sets). The final configuration was selected as the one that attained the best results in the cross-validation rolling-window process. All models used the profession and module as categorical features, and the day of the month, day of the week, month and year as covariates. The inclusion of COVID-19-related covariates and information on the *Safe Delivery App* training for healthcare professionals was tested but did not result in any clear improvement in the model accuracy.

In general, covariates can be forecast to simulate future epidemic outbreaks, among other events, or plan app workshops, as potential use cases. In this context, the future usage of the app depends on not only the past usage but also the other features that are incorporated in the models.

Regarding the specifications of each method, the SARIMA forecasting was performed using the auto-ARIMA functionality, which means that all combinations of regular polynomials up to degree 5, seasonal polynomials up to degree 2, up to 2 regular and up to 1 seasonal difference were tried, using the Akaike information criterion to select the best of them. Our neural network with categorical embeddings had three fully connected layers with 1000, 500 and 1 cells; the activation functions for the first and second dense layers were ReLU and sigmoid, respectively, and we used the mean absolute error as the loss function and Adam as the optimization method. The best performing DeepAR model was found to be that using 20 2-layer LSTM cells, a negative binomial distribution, a dropout rate of 0.01, 300 epochs and a training batch size of 30. The selected GP-Copula variant had exactly the same settings, except that only five epochs were considered—as this method is considerably more computationally intensive and the model was already reaching convergence.

### 3.3 Results and discussion

**Fig. 2:**
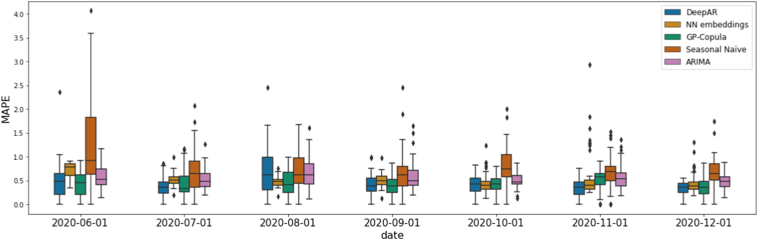
Mean absolute percentage error (MAPE) boxplot distribution from individual time series displayed by model and by prediction month.

Most of the forecasting models evaluated could capture the trend of the time series, with results differing mainly in the estimation of the daily patterns specific to each time series. The results are summarized in Table 1, which shows several error metrics averaged over across all monthly predictions: Mean Absolute Scaled Error (MASE), Mean Absolute Percentage Error (MAPE), Symmetric Mean Absolute Percentage Error (sMAPE), Root Mean Squared Error (RMSE) and Mean Squared Error (MSE) [76]. In Fig. 2 the boxplot distribution of the individual MAPE scores are displayed for each model and for each prediction month. We observe that recent months tend to have lower scores, partially due to the enlargement of the historical data used as the training sample. Note we are comparing two baselines (the very crude seasonal naïve and the classical ARIMA), with three methods incorporating deep learning elements.

**Table 1:** Error metrics for the different forecasting models evaluated. The final errors represent an average over the individual predictions using a rolling window from 2020-06-01 to 2020-12-01, with a forecasting horizon of 30 days.

Model	MASE	MAPE	sMAPE	RMSE	MSE
Seasonal Naïve	1.3221	0.7945	0.7294	8.2179	84.2975
ARIMA	0.8752	0.5569	0.7416	4.5020	21.2377
NN w/categorical embeddings	1.0381	0.5283	0.6547	12.4945	250.0235
DeepAR	0.8110	0.4373	0.5689	4.4461	22.1728
GP-Copula	0.8209	0.4285	0.6564	4.4088	21.5326

**Fig. 3:**
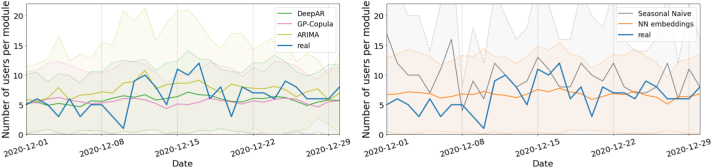
Real vs. forecasted values for the *Infection Prevention* module demand by *midwifes* on December 2020. The predictions and 50 % confidence intervals of the DeepAR, GP-Copula, ARIMA, NN with categorical embeddings and seasonal naïve models are shown.

**Fig. 4:**
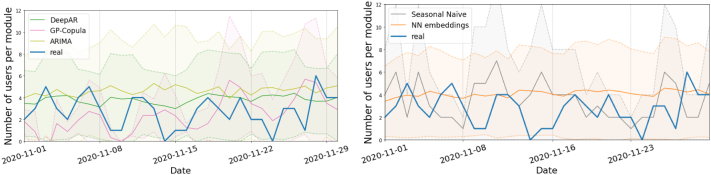
Real vs. forecasted values for the *Active Management of Third Stage Labour* module demand by *physicians* on November 2020. The predictions and 50 % confidence intervals of the DeepAR, GP-Copula, ARIMA, NN with categorical embeddings and seasonal naïve models are shown.

Fig. 3, Fig. 4 show an example of the forecasts for each model. While the performance of the DeepAR and GP-Copula methods is similar (left panel), the former shows a tighter 50 % confidence prediction interval that fits better the shape of the actual series. The seasonal ARIMA model also produces remarkably accurate predictions, which accounts for its extended use at present even though more sophisticated methods are available, although it shows a larger forecast uncertainty (as shown by the wider confidence interval). The forecasts for the other evaluated models are displayed in the right panel. The performance of the GP-Copula model trained over only five epochs is comparable to that of the DeepAR model trained over 300 epochs, though it still needs more time and resources. Consequently, DeepAR would be the preferred option in a production environment. However, if higher accuracy were critical and there were no constraints on computational time and resources, the use of GP-Copula with an increased number of epochs would be justified.

Other models such as Facebook's Prophet [77] and DeepVAR (the simplest multivariate extension of DeepAR) [73], both with their default settings, were also tested; however, their performance was inferior to that of the ARIMA benchmark, and thus, they were not included in the analysis.

From the medical point of view, the applicability of this approach is straightforward with similar kinds of e-learning mHealth applications. Some examples include Leap [78], an e-learning mHealth app with services in Kenya to train healthcare workers [79], [80], and Jibu [81] that supports nurses and midwives through e-learning in Kenya and other Sub-Saharan African countries. The solution proposed in this research is general enough to adapt to any content structure for any app or platform, provided that user-content interaction logs are recorded, thus it can be easily reproduced for other similar applications where in-app components interaction behavior is recorded. Moreover, this approach could also be applied to any other kind of demand forecasting, not necessarily related to e-learning, such as online medical consultation demand.

Regarding privacy concerns, data is fully anonymized as this approach is centered around user behavior, and in particular, the healthcare worker interaction with the e-learning content of the app. Therefore, only user characteristics such as country, region, profession or workplace are recorded. However, this work will still be reproducible even if that information was not available, as the content demand forecast could be always estimated and instead of dividing users by a given user characteristic (e.g. profession) they could be grouped by any other type of profiling technique (e.g. by their connection frequency, length of time using the app since first login, number of unique content checked, etc.); leading towards our aim of content personalization.

Other external information such as training programs, epidemic outbreaks, could be considered sensitive, however, this information could also be encoded to make it anonymized.

There are no available e-learning public datasets that we are aware of that could be used to assess the reproducibility and generalizability of our findings in the real world. Tools that can assist in the generation of synthetic data that can realistically simulate in-app activity are a step in the right direction. For example, the Open Synthetic Data Generator [82] can be used to fabricate data resembling the logs produced by a heterogeneous user base employing an app with e-learning and e-commerce functionalities.

## 4 Summary and conclusion

Overall, we found that the DeepAR and GP-Copula deep learning models are the most accurate for daily forecasting of the content demand. This result holds across different contents (modules) and user types (professions), as these two models show less error variability in the overall results of each individual time series.

Although the evaluated dataset corresponds to India, the proposed methodology can be applied to different countries or geographical areas and to additional contents. DeepAR constitutes a generalizable model that can correctly capture the trend behavior of the time series and anticipate user demand for a particular content depending on the user profile. We provided a solution that can be used in operational settings to obtain real-time demand estimates, due to the flexibility and speed of the model implementation.

The proposed approach can provide valuable insights into user behavioral profiles for different types of e-learning content, thereby enabling personalization of the mHealth app. In addition to e-learning, the proposed approach can be applied to other types of mHealth digital applications to identify the parts of the apps with more usage demand; these findings can then be used to personalize the in-app utilities for users.

Moreover, this work demonstrates a novel application in which the outcomes of health content demand forecasting can be used to simulate and test the delivery of interventions to support health service personnel in planning training programmes and content for healthcare professionals according to the relevant user profiles and needs. The findings can help (1) develop more effective plans and designs for incentivization campaigns to promote the user engagement with the content and (2) estimate the content usage trends for different user profiles to facilitate personalization. Personalized and targeted training content for health personnel can help build capacity and promote public health, especially in resource-poor regions.

## Data and code

All data used in this analysis comes from the Safe Delivery App logs and belongs to the Maternity Foundation. For inquiries regarding its use, please contact them at mail@maternity.dk.

Code used to generate the predictions discussed in this paper is publicly available from github at: https://github.com/benshi-ai/paper_2021_kdd_dshealth.

## References

[1] Hosny A., Aerts H.J. (2019). Artificial intelligence for global health. Science.

[2] Wahl B., Cossy-Gantner A., Germann S., Schwalbe N.R. (2018). Artificial intelligence (AI) and global health: how can AI contribute to health in resource-poor settings?. BMJ Glob Health.

[3] O’Connor S. (2018). Big data and data science in health care: what nurses and midwives need to know. J Clin Nurs.

[4] Marsch L.A. (2021). Digital health data-driven approaches to understand human behavior. Neuropsychopharmacology.

[5] U. N. P. Fund (2020). Costing the three transformative results.

[6] U. N. I. G. for Child Mortality Estimation (2020).

[7] UNICEF, U. N. P. F. World Health Organization, T. W. Bank (2019).

[8] Nove A., Friberg I.K., de Bernis L., McConville F., Moran A.C., Najjemba M., ten Hoope-Bender P., Tracy S., Homer C.S. (2021). Potential impact of midwives in preventing and reducing maternal and neonatal mortality and stillbirths: a lives saved tool modelling study. Lancet Glob Health.

[9] UNICEF, C. T. C. S. WHO (2015).

[10] Lawn J.E., Blencowe H., Waiswa P., Amouzou A., Mathers C., Hogan D., Flenady V., Frøen J.F., Qureshi Z.U., Calderwood C. (2016). Stillbirths: rates, risk factors, and acceleration towards 2030. Lancet.

[11] M. Foundation (2022). Safe delivery app. https://www.maternity.dk/safe-delivery-app/.

[12] M. Foundation (2022). Maternity foundation. https://www.maternity.dk/.

[13] Xu S., Chan H.K. (2019). Forecasting medical device demand with online search queries: a big data and machine learning approach. Procedia Manuf.

[14] Guitart A., Chen P.P., Bertens P., Periáñez Á. (2018). 2018 IEEE conference on future of information and communication conference (FICC).

[15] del Río A.F., Guitart A., Periáñez Á. (2021). Intelligent data analysis.

[16] Lund S., Boas I.M., Bedesa T. (2016). Association between the safe delivery app and quality of care and perinatal survival in Ethiopia: a randomized clinical trial. JAMA Pediatr.

[17] Olusola Oladeji M.T., Oladeji B. (2022). Strengthening quality of maternal and newborn care using catchment based clinical mentorship and safe delivery app: a case study from somali region of ethiopia. Int J Midwifery Nurs Pract.

[18] Marsch L.A. (2021). Digital health data-driven approaches to understand human behavior. Neuropsychopharmacology.

[19] Buckeridge D.L. (2020). Precision, equity, and public health and epidemiology informatics–a scoping review. Yearb Med Inform.

[20] Overdijkink S.B., Velu A.V., Rosman A.N., Van Beukering M.D., Kok M., Steegers-Theunissen R.P. (2018). The usability and effectiveness of mobile health technology–based lifestyle and medical intervention apps supporting health care during pregnancy: systematic review. JMIR Mhealth Uhealth.

[21] Dolley S. (2018). Big data’s role in precision public health. Front Public Health.

[22] Dowell S.F., Blazes D., Desmond-Hellmann S. (2016). Four steps to precision public health. Nature.

[23] Hosny A., Aerts H.J. (2019). Artificial intelligence for global health. Science.

[24] Wahl B., Cossy-Gantner A., Germann S., Schwalbe N.R. (2018). Artificial intelligence (AI) and global health: how can AI contribute to health in resource-poor settings?. BMJ Glob Health.

[25] Forero R., Nahidi S., De Costa J., Mohsin M., Fitzgerald G., Gibson N., McCarthy S., Aboagye-Sarfo P. (2018). Application of four-dimension criteria to assess rigour of qualitative research in emergency medicine. BMC Health Serv Res.

[26] Dunleavy G., Nikolaou C.K., Nifakos S., Atun R., Law G.C.Y., Car L.T. (2019). Mobile digital education for health professions: systematic review and meta-analysis by the digital health education collaboration. J Med Internet Res.

[27] Walsh K. (2014). The future of e-learning in healthcare professional education: some possible directions. Ann Ist Super Sanita.

[28] Olaniyi B.Y., del Río A.F., Periáñez Á., Bellhouse L. (2022). Proceedings of 2022 ACM KDD workshop on applied data science for healthcare (DSHealth 2022).

[29] Guitart A., Heydari A., Olaleye E., Ljubicic J., del Río A.F., Perián˜ez Á., Bellhouse L. (2021). NeurIPS machine learning in public health workshop (MLPH 2021).

[30] Katsaris I., Vidakis N. (2021). Adaptive e-learning systems through learning styles: a review of the literature. Adv Mob Learn Educ Res.

[31] Anantharaman H., Mubarak A., Shobana B. (2018). 2018 IEEE conference on e-learning, e-management and e-services (IC3e).

[32] Durga S., Hallinan S., El-Nasr M.S., Shiyko M., Sceppa C. (2021).

[33] Bargen T., Zientz C., Haux R. (2014). Gamification for mhealth–a review of playful mobile healthcare. Integr Inf Technol Manag Qual Care.

[34] Periáñez Á., Saas A., Guitart A., Magne C. (2016). 2016 IEEE international conference on data science and advanced analytics (DSAA), IEEE, Montreal, Canada.

[35] Bertens P., Guitart A., Chen P.P., Periáñez Á. (2018). 2018 IEEE conference on computational intelligence and games (CIG).

[36] del Río A.F., Chen P.P., Periáñez Á. (2019). 2019 IEEE conference on games (CoG), IEEE.

[37] Qiu F., Zhang G., Sheng X., Jiang L., Zhu L., Xiang Q., Jiang B., Chen P.-K. (2022). Predicting students’ performance in e-learning using learning process and behaviour data. Sci Rep.

[38] Alshehri M., Cristea A.I. (2022). International conference on artificial intelligence in education.

[39] Alshehri M., Alamri A., Cristea A.I., Stewart C.D. (2021). Towards designing profitable courses: predicting student purchasing behaviour in moocs. Int J Artif Intell Educ.

[40] Waheed H., Hassan S.-U., Aljohani N.R., Hardman J., Alelyani S., Nawaz R. (2020). Predicting academic performance of students from vle big data using deep learning models. Comput Hum Behav.

[41] Fotso J.E.M., Batchakui B., Nkambou R., Okereke G. (2022). Artificial intelligence for data science in theory and practice.

[42] Shou Z., Chen P., Wen H., Liu J., Zhang H. (2022). Mooc dropout prediction based on multidimensional time-series data. Math Probl Eng.

[43] Chen M., Wu L. (2021). Journal of physics: conference series.

[44] Zheng Y., Gao Z., Wang Y., Fu Q. (2020). Mooc dropout prediction using fwts-cnn model based on fused feature weighting and time series. IEEE Access.

[45] Tang C., Ouyang Y., Rong W., Zhang J., Xiong Z. (2018). International conference on artificial intelligence in education.

[46] Chen F., Cui Y. (2020). Utilizing student time series behaviour in learning management systems for early prediction of course performance. J Learn Anal.

[47] Nguyen V.A., Nguyen Q.B., Nguyen V.T. (2018). Proceedings of the 2nd international conference on E- Society, E-Education and E-Technology.

[48] Jo Y., Maki K., Tomar G. (2018).

[49] Ravichandran M., Kulanthaivel G. (2015). 2015 international conference on computing and communications technologies (ICCCT).

[50] Namoun A., Alshanqiti A. (2020). Predicting student performance using data mining and learning analytics techniques: a systematic literature review. Appl Sci.

[51] Soyiri I.N., Reidpath D.D. (2013). An overview of health forecasting. Environ Health Prev Med.

[52] Liu C., Wang W.Y.C., Khan G. (2021). 2021 5th international conference on medical and health informatics.

[53] Chen W., Yu L., Li J. (2021). Forecasting teleconsultation demand with an ensemble attention-based bidirectional long short-term memory model. Int J Comput Intell Syst.

[54] Kazmi S., Bozanta A., Cevik M. (2021). Time series forecasting for patient arrivals in online health services.

[55] Huang Y., Xu C., Ji M., Xiang W., He D. (2020). Medical service demand fore- casting using a hybrid model based on arima and self-adaptive filtering method. BMC Med Inform Decis Mak.

[56] Mburu S., Oboko R. (2018). A model for predicting utilization of mHealth interventions in low-resource settings: case of maternal and newborn care in kenya. BMC Med Inform Decis Mak.

[57] Motwani A., Shukla P.K., Pawar M., Kumar M., Ghosh U., Alnumay W., Nayak S.R. (2023). Enhanced framework for covid-19 prediction with computed tomography scan images using dense convolutional neural net- work and novel loss function. Comput Electr Eng.

[58] Divya V., Krishnan S.S.K.V.G., Kumar M. (2023). Signal conducting system with effective optimization using deep learning for schizophrenia classification. Comput Syst Sci Eng.

[59] Singh M., Shrimali V., Kumar M. (2022). Detection and classification of brain tumor using hybrid feature extraction technique. Multimed Tools Appl.

[60] Kumar S., Gupta S.K., Kumar V., Kumar M., Chaube M.K., Naik N.S. (2022). Ensemble multimodal deep learning for early diagnosis and accurate classification of covid-19. Comput Electr Eng.

[61] Taylor K., Dangerfield B. (2005). Modelling the feedback effects of reconfiguring health services. J Oper Res Soc.

[62] Eswaran C., Logeswaran R. (2012). A dual hybrid forecasting model for support of decision making in healthcare management. Adv Eng Softw.

[63] Alexandrov A., Benidis K., Bohlke-Schneider M., Flunkert V., Gasthaus J., Januschowski T., Maddix D.C., Rangapuram S., Salinas D., Schulz J. (2020). Gluonts: probabilistic and neural time series modeling in python. J Mach Learn Res.

[64] Chollet F. (2015). Keras. https://github.com/fchollet/keras.

[65] Chen T., Li M., Li Y., Lin M., Wang N., Wang M., Xiao T., Xu B., Zhang C., Zhang Z. (2015).

[66] Hyndman R., Khandakar Y. (2008). Automatic time series forecasting: the forecast package for r. J Stat Softw.

[67] Box G.E.P., Jenkins G. (1990).

[68] LeCun Y., Bengio Y., Hinton G. (2015). Deep learning. Nature.

[69] Guo C., Berkhahn F. (2016). https://arxiv.org/abs/1604.06737.

[70] Salinas D., Flunkert V., Gasthaus J., Januschowski T. (2020). Deepar: probabilistic forecasting with autoregressive recurrent networks. Int J Forecast.

[71] Benidis K., Rangapuram S.S., Flunkert V., Wang Y., Maddix D., Turkmen C., Gasthaus J., Bohlke-Schneider M., Salinas D., Stella L., Aubet F.-X., Callot L., Januschowski T. (2022). Deep learning for time series forecasting: tutorial and literature survey. ACM Comput Surv.

[72] Papastefanopoulos V., Linardatos P., Kotsiantis S. (2020). Covid-19: A comparison of time series methods to forecast percentage of active cases per population. Appl Sci.

[73] Salinas D., Bohlke-Schneider M., Callot L., Medico R., Gasthaus J., Wallach H., Larochelle H., Beygelzimer A., d'Alch´e-Buc F., Fox E., Garnett R. (2019). Advances in neural information processing systems.

[74] Racine J. (2000). Consistent cross-validatory model-selection for dependent data: hv-block cross-validation. J Econ.

[75] Cerqueira V., Torgo L., Mozetiˇc I. (2020). Evaluating time series forecasting models: an empirical study on performance estimation methods. Mach Learn.

[76] Botchkarev A. (2019). A new typology design of performance metrics to measure errors in machine learning regression algorithms. Interdiscip J Inf Knowl Manag.

[77] Taylor S.J., Letham B. (2018). Forecasting at scale. Am Stat.

[78] Africa A.H. (2022). Leap: the mHealth platform. Our solution. https://www.leaphealthmobile.com/our-solution.

[79] Abajobir A., de Groot R., Wainaina C., Njeri A., Maina D., Njoki S., Mbaya N., Donfouet H.P.P., Pradhan M., Janssens W. (2021). The impact of i-push on maternal and child health care utilization, healthoutcomes, and financial protection: study protocol for a cluster randomized controlled trial based on financial and health diaries data. Trials.

[80] Aerts A., Bogdan-Martin D. (2021). Leveraging data and ai to deliver on the promise of digital health. Int J Med Inform.

[81] Africa A.H. (2022). Jibu. https://jibu.africa/.

[82] benshi.ai (2022). Open synthetic data generator. https://github.com/benshi-ai/open-synthetic-data-generator.

